# Health effects of selected trace elements in hairdressing cosmetics on hairdressers in Enugu, Nigeria

**DOI:** 10.1038/s41598-021-00022-1

**Published:** 2021-10-13

**Authors:** Onyenekenwa Cyprian Eneh

**Affiliations:** Institute for Development Studies, Enugu Campus, University of Nigeria, Nsukka, Nigeria

**Keywords:** Biochemistry, Environmental sciences, Health care, Chemistry

## Abstract

Could trace elements in hairdressing cosmetics prove carcinogenic to hairdressers, who use them regularly as raw materials in hairdressing business? The toxic effects of selected trace elements contained in hairdressing cosmetics on hairdressers were investigated by regressing the quality of life values for hairdressers with their blood/urine levels of the toxic metals A hundred subjects were randomly drawn from a cluster of hairdressers. Blood lead level, 17.47 ± 4.59 µg/dL, confirmed that the hairdressers were regularly exposed to lead. Mean blood mercury level, 25.06 ± 4.11 ng/dL, was above the normal blood mercury concentration of less than 10–20 ng/mL. Mean blood cadmium level, 3.64 ± 1.87 µg/dL, was within the normal range, < 5.0 µg/dL. Mean urine arsenic level, 96.16 ± 38.34 µg/L, was above normal range, ≤ 50 μg/dL, but within high normal range, > 50 to < 200 μg/dL. Blood nickel level, 0.49 ± 0.20 µg/dL, was above both the healthy range, 0.14–0.65 µg/dL, and the most reliable value of 0.2 µg/dL. Negative correlations existed between the quality of life and levels for lead (R = − .017), mercury (R = − .008), arsenic (R = − .072) and nickel (R = − .168), but there was positive correlation with cadmium level (R = .123). At 0.096, the probability value (*p* value) for nickel was significant at 10%. *P* value as insignificant at 1%, 5% and 10% for lead (0.868), mercury (0.940), cadmium (0.224) and arsenic (0.475). Therefore, Ni contained in hairdressing cosmetics might have carcinogenic effects that affected the quality of life of hairdressers, unlike other trace elements tested, namely, lead, mercury, cadmium and arsenic. Further research with the tumor markers assessment is recommended to confirm the carcinogenic effect of Ni.

## Introduction

### Trace elements in hairdressing cosmetics and their carcinogenic effects

Trace elements commonly incorporated in hairdressing cosmetics are lead (Pb), mercury (Hg), cadmium (Cd), arsenic (As) and nickel (Ni). They are toxic and poison the body when accumulated in tissues^1^. Pb is absorbed through skin contact, ingested (swallowed) or inhaled as particles. Once accumulated in the body, Pb is distributed to major organs (brain, kidneys, liver and bones/teeth). Absorption of Pb is highest through inhalation than through the skin and ingestion. In human body, Pb stores in bones, blood, and tissues for continual internal exposure^2^. Blood lead level is a measure of the blood lead concentration (µg/dL). High Pb levels indicate its intoxication or poisoning^3^. Lead poisoning may lead to high blood pressure, joint/muscle pain, difficulty with memory/concentration, headache, abdominal pain, mood disorder, miscarriage, premature birth, low birth weight, and slowed growth in children. It has its global tolls, accounting for 1.06 million deaths in 2017. In 2016, it accounted for 63.2% of intellectual disability, 10.3% of hypertensive heart disease, 5.6% of heart disease and 6.2% of stroke^4–6^. If symptoms occur with concentrations lower than 80 µg/dL, a need for treatment is indicated. As a highly toxic element, there is no known safe level of lead exposure^7^.

Hg, which is a liquid at room temperature, readily vaporizes into the air to be easily inhaled. This is the commonest way of exposure of humans to Pb and the most harmful to health. Pregnant women are particularly sensitive to harmful effects of Hg which can pass from a mother’s body to the developing foetus and can pass through breast milk to nursing infants. Hair, urine or blood test can detect Hg exposure or poisoning or intoxication. Hg poisoning may manifest in shortness of breath, cough, chest pain, sore throat, diarrhoea, nausea, vomiting, a metallic taste in the mouth, increase in blood pressure or heart rate, headache, eye irritation and vision problems. Other symptoms are insomnia, loss of appetite, fatigue, shaking movements in one or more parts of the body (tremors), forgetfulness, and changes in vision and hearing^8,9^.

Cd is a very poisonous element that forms a variety of chelates lacking in recycling techniques. By decreasing density, volume and number of sperms, and increasing immature sperm forms, Cd has the potential to affect reproduction^10–12^. Acute As poisoning affects the lungs, skin, kidneys, and liver. Its symptoms include diarrhoea, vomiting, vomiting blood, blood in the urine, cramping muscles, hair loss, stomach pain, convulsions and more. Breathing dust containing Ni may manifest in chronic bronchitis, reduced lung function, and cancer of the lung and nasal sinus. Ni poisoning may manifest in headache, nausea, vomiting, dizziness, irritability, and difficulty sleeping^13,14^ (Mayo Clinic 2012; Agency for Toxic Substances and Disease Registry 2005).

Kilic et al.^15^ analyzed the levels of some trace elements (copper, Cu; cobalt, Co; lead, Pb; nickel, Ni; chromium, Cr; cadmium, Cd and arsenic, As) in 19 different home-made cosmetic products by inductively coupled plasma mass spectrometry (ICP-MS) after microwave digestion. The detected levels of the elements in the cosmetic products were higher than the standards set by the World Health Organization (WHO). The rank order of the elements based on THQ index was established as Cr > Pb > Ni. The potential non-carcinogenic limit was considerably lower than the safe risk limit (THQ > 1). The study was of limited practical significance because it did not interrogate the carcinogenic effects of the toxic trace elements on the users of the home-made cosmetic products and their effects on the quality of life of the participants, but merely assessed the levels of the concentrations of the trace elements, apparently to compare them with WHO standards to form the basis of advice on quality control. It did not address the gap in literature, as to whether the trace elements in hairdressing cosmetics could prove carcinogenic to hairdressers who use the hairdressing cosmetics regularly as the main raw materials in the hairdo industry. Therefore, this study interrogated the toxic effects of selected trace elements contained in hairdressing cosmetics on hairdressers and their effects on the quality of life of the users by regressing the quality of life with blood/urine levels of the toxic metals.

### Quality of life (QOL)

QOL represents the general well-being of individuals and societies. It refers to the negative and positive features of life and the expectations of an individual or society for a good life^16^.

Heavy metals poisoning affects quality of life. The symptoms of poisoning of heavy metals contained in hairdressing cosmetics can be summarized as follows:

#### Lead (Pb)

High blood pressure, joint pain, muscle pain, poor memory, poor concentration, headache, abdominal pain, mood disorder, miscarriage, premature birth, low birth weight, and slow child growth.

#### Mercury (Hg)

Shortness of breath, cough, chest pain, sore throat, diarrhoea, nausea, vomiting, metallic taste in the mouth, increase in blood pressure, headache, eye irritation, poor vision problems, insomnia, loss of appetite, fatigue, shaking movements in one or more parts of the body (tremors), forgetfulness, poor vision and poor hearing.

#### Cadmium (Cd)

Childlessness.

#### Arsenic (As)

Diarrhoea, vomiting, vomiting blood, blood in the urine, cramping muscles, hair loss, stomach pain, and more convulsions.

#### Nickel (Ni)

Headache, nausea, vomiting, dizziness, irritability, and difficulty sleeping.

### Study aim and objectives

Since these trace elements are commonly incorporated in hairdressing cosmetics, which are common raw materials in the hairdressing industry, it is necessary to ascertain their carcinogenic effects on hairdressers. In this study, the quality of life of hairdressers, as estimated by 36 symptoms of poisoning of the trace elements commonly incorporated in hairdressing cosmetics (which could be absorbed by dermal contact, inhaled or swallowed) was correlated with blood/urine levels of the trace elements.

## Methods

### Area of study

Enugu in Enugu State in Southeast of Nigeria is populated by Igbos—one of the most populous ethnic nationalities in Nigeria. The coal city has a population of 722,664^17^. It was the capital of the defunct Eastern Region from Nigeria’s independence in 1960 until January 15, 1970, when the Nigeria-Biafra civil war ended^18^. Enugu became the capital city of the East Central State in 1970 and retained the capital city status of the old Anambra State that emerged from dividing the East Central State in 1976 into Anambra and Imo States. It also retained the capital city status for Enugu State in 1991 when Anambra State was split into Enugu State and Anambra State. Again it retained the capital city status for Enugu State when in 1996 Ebonyi State was carved out of the old Enugu State^19^.

Enugu city consists of neighbourhoods, which have been classified according to their population and population density levels that characterize layouts. These are the high-, medium- and low-density neighbourhoods. High-density neighbourhood harbours upwards of 700 persons per hectare. About 70% of the city’s population live in low-income (20,000/50,000 per month) tenements. Medium-density neighbouroods house about 28% of the city population in blocks of flats rented at 10,000–20,000/monthly with average population density of 350–400 persons per hectare. Household size of 6–6.5 persons has been observed. This is where New Haven belongs. Low-density neighbourhood has about 2% of the city population and covers about 20% of the city area. Low density of 18–60 persons per hectare, storey-buildings, bungalows, blocks of flats and semi-detached duplexes characterize the area^19^.

### Study design and procedure

Nanka Street in New Haven in Enugu metropolis in Southeast Nigeria is known for a colony of hairdressing salons, always busy every day of the week and late into the night. This industrial cluster involving mostly women formed the target of the study. Convenience sampling technique, based on participant availability, willingness to participate and meeting the inclusion criteria, was adopted. A total of 100 hairdressers (proprietors, workers and apprentices) participated in the study. Inclusion criteria were upwards of 18 years of age and upwards of 6-month prior-study engagement in the job. Exclusion criteria were less than 18 years of age and less than 6-month engagement in the job.

The procedure for the study was explained to the subjects from whom informed consent for participation in the study and academic publication/s of the results of the study with anonymity were sought and obtained. Unused sterile needles, syringes and blood sample bottles, sourced from a tertiary health institution that had imported them and other pharmaceuticals and medical equipment from recognized reputable pharmaceutical companies in China and United States of America (USA) and considered to be trace elements-free, were used by a phlebotomist (staff of the health institution) to collect blood samples from the vein of each participant into the sterile sample bottle, with swab cotton wool and methylated spirit. Where necessary, test tubes and containers were sterilized by autoclaving and use of 70% alcohol before use. Each participant was given a sterilized sample bottle to supply early morning urine, with instruction to store the sample in the fridge between collection and delivery to the phlebotomist. A Medical Laboratory Scientist working in the health institution tested the samples for blood Pb, Hg, Cd and Ni levels and the urine levels of As (independent variables).

Thirty-six (36) common symptoms of poisoning of the trace elements (which could be absorbed by dermal contact, inhaled or swallowed) were used to estimate the quality of life of participants and correlated with blood Pb, Hg, Cd levels and Ni and urine level of As. These symptoms were high blood pressure, joint pain, muscle pain, poor concentration, headache, abdominal pain, mood disorder, short breath, cough, chest pain, sore throat, diarrhea, nausea, vomiting, metal taste in the mouth, eye irritation, insomnia, poor vision, loss of appetite, fatigue, tremors, forgetfulness, poor hearing, childlessness, vomiting blood, blood in urine, cramping muscles, hair loss, stomach pain, convulsions, dizziness, irritability, miscarriage, premature birth, low birth weight, and slow child growth.

Testing for As, Pb, and Cd followed the Environmental Protection Agency (EPA) of the United States of America (USA) Method—200_13—Trace element determination via Atomic Absorption Graphite Furnace Spectrometer using Buck Scientific Atomic Absorption Spectrophotometer (GFAAS, made in USA). Pd-Mg mixture was served as the matrix modifier for As, while Ni was used as matrix modifier for Pb and Cd^20^.

Testing for Hg used absorption spectrometry (dithizone colorimetry), neutron activation analysis or cold vapor atomic absorption spectrometry of the Environmental Protection Agency (EPA) of the United States of America (USA) Method—200_13—Trace element determination via Atomic Absorption Graphite Furnace Spectrometer using Buck Scientific Atomic Absorption Spectrophotometer (GFAAS, made in USA)^20^.

To determine Ni, 2-[(2-mercaptophenylimino)methyl]phenol (MPMP) was used to form a brown 1:2 MPMP-nickel(II) complex at pH > 10, which was extracted into chloroform. The complex had a maximum absorption at 421 nm. The relative standard deviation at 0.018 µg mL-1 is 1.1% (n = 8)^21^.

The Health Research and Ethical Review Committee of the University of Nigeria Teaching Hospital (UNTH), Ituku-Ozalla, Enugu gave the ethical approval for the study. Methods followed the relevant guidelines and regulations.

The data were subjected to descriptive statistics to determine the means and standard deviations of the serum/urine levels of the trace elements. Pearson correlation analysis was used to determine the relationship between the variables: serum levels of the toxic metals in hairdressing cosmetics (independent) and quality of life of hairdressers (dependent). Analysis was performed using Statistical Package for Social Sciences (SPSS) Version 23.

### Ethical approval

The Health Research and Ethical Review Committee of the University of Nigeria Teaching Hospital (UNTH), Ituku-Ozalla, Enugu gave the ethical approval for the study.

### Consent to participate

All subjects gave informed consent to participate in the study.

## Results and discussion

Table 1 shows the demographics of subjects.

**Table 1:** Demographics of subjects. *Source*: Field work 2020.

Demographics	Frequency/percentage
**Sex**	
Male	0
Female	100
**Age (years)**	
< 30	13
30–50	73
51–70	12
> 70	2
**Highest educational qualification**	
Informal	0
Primary	3
Secondary	72
OND	14
BSc/BA/HND	11
> BSc/BA/HND	0
**Marital status**	
Single	67
Married	29
Divorced/separated	4
**No. of years in career**	
< 5 years	2
6–10 years	64
11–20 years	26
21–30 years	6
31–40 years	2
> 40 years	0

Table 1 shows that all subjects (100%) were adult females. Participants were aged < 30 (13%), 30–50 years (73%), 50–70 years (12%) and > 70 years (2%). Participants were all formally educated, with 3% having primary education, 72% having secondary education, 14% possessing Ordinary National Diploma (OND), and 11% possessing BSc/BA/HND certificates. Marital statuses of participants were mostly single (67%), married (29%), and divorced or separated (4%). Work duration of participants were 2% for < 5 years, 64% for 6–10 years, 26% for 11–20 years, 6% for 21–30 years, 2% for 31–40 2 years, and none for above 40 years.

The mode age bracket of 30–50 (73%), formal education for all participants (3% with primary education, 72% with secondary education, 14% with OND and 11% with BSc/BA/HND) and the single marital status of most participants (67%) confirmed earlier reports. Eneh^22^ reported that only 8% of respondent entrepreneurs and technical service providers were < 30 years of age, 63% were aged 31–50 years, 27% were aged 51–70 years; and 2% were > 70 years of age. Young unmarried people were keeping away from technical apprenticeship and services provision as careers. Participant entrepreneurs had joined the career decades earlier and new entrants hardly tricked in. Artisans were retiring too early (51–70 years of age) because of lack of hands as apprentices and young workers. Subjects were all formally educated and mostly literate with 72% having secondary education and 11% with Bachelor degree and its equivalents. Eneh^23^ opines that with the introduction of Universal Basic Education (UBE) in 1999 in Nigeria, education expansion and access have enhanced literacy. With rising unemployment rates and unemployability of Nigerian graduates and school-leavers/drop-outs, marriage has become a secondary project for Nigerian youths^24,25^.

Table 2 shows the quality of life of hairdressers.

**Table 2:** Quality of life of hairdressers. *Source*: Field work 2020.

Symptom	Frequency/percentage	
1. High blood pressure:	Yes	3
	No	97
2. Joint pain:	Yes	14
	No	86
3. Muscle pain:	Yes	11
	No	89
4. Poor concentration:	Yes	15
	No	85
5. Headache:	Yes	82
	No	18
6. Abdominal pain:	Yes	9
	No	91
7. Mood disorder:	Yes	6
	No	94
8. Short breath:	Yes	2
	No	98
9. Cough:	Yes	35
	No	65
10. Chest pain:	Yes	26
	No	74
11. Sore throat:	Yes	36
	No	64
12. Diarrhoea:	Yes	19
	No	81
13. Nausea:	Yes	5
	No	95
14. Vomiting:	Yes	1
	No	99
15. Metallic taste in mouth:	Yes	46
	No	54
16. Eye irritation:	Yes	17
	No	83
17. Insomnia:	Yes	59
	No	41
18. Poor vision:	Yes	23
	No	77
19. Loss of appetite:	Yes	27
	No	73
20. Fatigue:	Yes	69
	No	31
21. Tremors (shaking movements in one or more parts of the body):	Yes	7
	No	93
22. Forgetfulness:	Yes	32
	No	68
23. Poor hearing:	Yes	16
	No	84
24. Childlessness:	Yes	13
	No	87
25. Vomiting blood:	Yes	1
	No	81
26. Blood in the urine:	Yes	29
	No	61
27. Cramping muscles:	Yes	38
	No	62
28. Hair loss:	Yes	86
	No	14
29. Stomach pain:	Yes	91
	No	9
30. Convulsions:	Yes	3
	No	97
31. Dizziness:	Yes	44
	No	56
32. Irritability:	Yes	69
	No	21
33. Miscarriage:	Yes	8
	No	92
34. Premature birth:	Yes	1
	No	99
35. Low birth weight:	Yes	2
	No	98
36. Slow child growth:	Yes	4
	No	96

Table 2 shows that subjects had high blood pressure (3%), joint pain (14%), muscle pain (11%), poor concentration (15%), headache (82%), abdominal pain (9%), mood disorder (6%), short breath (2%), cough (35%), chest pain (26%), sore throat (36%), diarrhoea (19%), nausea (5%), vomiting (1%), metal taste in the mouth (46%), eye irritation (17%), insomnia (59%), poor vision (23%), loss of appetite (27%), fatigue (69%), tremors (7%), forgetfulness (32%), poor hearing (16%), childlessness (13%), vomiting blood (1%), blood in urine (29%), cramping muscles (38%), hair loss (86%), stomach pain (91%), convulsions (3%), dizziness (44%), irritability (39%), miscarriage (8%), premature birth (1%), low birth weight (2%), and slow child growth (4%).

Table 3 shows the mean blood Pb, Hg, Cd and Ni, and urine As levels of subjects.

**Table 3:** Blood lead, mercury, cadmium and nickel, and urine arsenic levels of subjects. *Source*: SPSS version 16.

	Mean	SD	N
Pb (µg/dL)	17.4723	4.58927	100
Hg (µg/dL)	25.9628	4.11188	100
Cd (µg/dL)	3.6421	1.87017	100
As (µg/L)	96.1608	38.33936	100
Ni (µg/dL)	.4873	.20257	100

Table 3 shows that the mean blood levels were 17.47 ± 4.59 µg/dL for Pb, 25.06 ± 4.11 ng/mL for Hg, 3.64 ± 1.87 µg/dL for Cd and 0.49 ± 0.20 µg/L for Ni. Urine As level was 96.16 ± 38.34 µg/L.

Table 4 shows that QOL was negatively correlated with Pb (R = − .017), Hg (R = − .008), As (R = − .072) and Ni (R = − .168) level, but was positively correlated with Cd (R = .123) level. The probability value (*p* value) was significant at 10% for Ni (0.096), but was insignificant at benchmark levels of 1%, 5% and 10% for Pb (0.868), Hg (0.940), Cd (0.224) and As (0.475).

**Table 4:** Correlation between blood lead, mercury, cadmium and nickel, and urine arsenic levels and the quality life of subjects. *Source*: SPSS version 16.

	Pb	Hg	Cd	As	Ni
**QOL**					
Pearson Correlation	− .017	− .008	.123	− .072	− .168
Sig. (2-tailed)	.868	.940	.224	.475	.096
N	100	100	100	100	100

The mean blood Pb level, 17.47 ± 4.59 µg/dL, was above the normal range, 0–10 µg/dL (or 0.48 micromoles per liter, µmol/L) in adults and above the range 10–25 µg/dL that signals regular exposure to Pb. A level of 80 µg/dL calls for treatment (Agency for Toxic Substances and Disease Registry 2000). Mean blood Hg level, 25.06 ± 4.11 µg/dL, was above the normal level, < 10–20 μg/dL, and 35 μg/dL level caused by long-term exposure to Hg vapour^7^.

The mean blood Cd level, 3.64 ± 1.87 μg/dL, was within the normal range, < 5.0 μg/dL (Agency for Toxic Substances and Disease Registry 2000). The mean urine As level, 96.16 ± 38.34 μg/L, was within the normal range, ≤ 50 μg/L, and high normal range, > 50 to < 200 μg/L^7^. Blood Ni level was 0.49 ± 0.20 µg/dL, within both the healthy range, 0.14–0.65 µg/dL, and 0.2 µg/dL, which is the most reliable value in adults^7^.

Table 4 shows the correlation between blood Pb, Hg, Cd and Ni, as well as urine As levels and the quality of life of subjects.

There were negative correlations between the quality of life of hairdressers and the blood/urine levels of the trace elements at R = − .017 for Pb, R = − .008 for Hg, R = − .072 for As, and R = − .168) for Ni. But, there was positive correlation with Cd level at R = .123. This observation might be related to secondhand-smoke (SHS), since smokers often accompanied patrons to the hairdressing salons and smoked while waiting^26^. The probability value (*p* value) was significant at 10% for Ni (0.096), but insignificant at benchmark levels of 1%, 5% and 10% for Pb (0.868), Hg (0.940), Cd (0.224) and As (0.475). Therefore, Ni contained in hairdressing cosmetics might have carcinogenic effects that affected the quality of life of hairdressers, unlike other trace elements tested, namely, lead, mercury, cadmium and arsenic.

## Conclusions and recommendations

The quality of life of hairdressers, who regularly use hairdressing cosmetics as raw materials in the hairdo industry, were regressed against the blood/urine levels of trace elements commonly incorporated in hairdressing cosmetics in order to ascertain the carcinogenic effects of the elements on the randomly selected product users. Findings confirmed that the hairdressers were regularly exposed to lead. The mean blood mercury level was above the normal blood mercury concentration and close to the level associated with long-term exposure to mercury vapour. The mean urine arsenic level was above the normal range, but within high normal range. Blood nickel level was above both the healthy range and the most reliable level. There were negative correlations between the quality of life and levels for lead, mercury, arsenic and nickel. But, there was positive correlation with cadmium level, perhaps due to second-hand-smoking. The probability values were insignificant for mercury, cadmium and arsenic, but significant for nickel. Therefore, Ni contained in hairdressing cosmetics might have carcinogenic effects that affected the quality of life of hairdressers, unlike other trace elements tested, namely, lead, mercury, cadmium and arsenic. Further research with the tumor markers assessment is recommended to confirm the carcinogenic effect of Ni.

## Data Availability

Materials and data embedded in this work are transparently available.

## Code availability

Materials and data are in Microsoft Word with custom code.
